# The influence of time of day on disruptive behaviours in middle school students during physical education classes

**DOI:** 10.3389/fspor.2025.1546436

**Published:** 2025-04-04

**Authors:** Mohamed Sami Bouzid, Hassan Melki, Ghazi Racil, Aymen Hawani, Sabra Hammoudi, Youssef Rezgani, Johnny Padulo

**Affiliations:** ^1^ Higher Institute of Sport and Physical Education, ISSEP Ksar-Saïd, Tunis, Tunisia; ^2^ Physical Activity, Sport and Health, Research Unit (UR18JS01), National Observatory of Sport, Tunis, Tunisia; ^3^ Research Laboratory (LR23JS01) “Sport Performance, Health & Society”, ISSEP Ksar-Saïd, Tunis, Tunisia; ^4^ Department of Biological Sciences, Higher Institute of Sport and Physical Education, ISSEP of Gafsa, Gafsa, Tunisia; ^5^ Department of Biomedical Sciences for Health, Università Degli Studi di Milano, Milan, Italy

**Keywords:** disciplinary incidents observing system, disruptive behaviour, physical education, morning vs. afternoon, classroom management

## Abstract

**Introduction:**

Understanding the factors influencing disruptive behaviour (DB) in Physical Education (PE) is essential for optimizing learning environments and student engagement. The aim of this study was to measure the frequency of DB in PE at two different times of the day, morning and afternoon, over an eight-week period.

**Methods:**

One hundred thirty-seven male students participated in the study, with a mean age of 13.63 ± 0.7 years and PE experience of 5.7 ± 0.3 years. The Disciplinary Incidents Observing System (DIOS) was used to measure the frequency of DB occurrences during PE sessions.

**Results:**

A significant difference was found in the average frequency of DBs observed between morning and afternoon sessions (*p* = 0.008 < 0.05). A total of 160 DBs were recorded during morning sessions, averaging 40 DBs per session or 0.8 DBs per minute. In contrast, 97 DBs were observed during afternoon sessions, averaging 24.25 DBs per session or 0.485 DBs per minute. These results suggest that physical activity in afternoon PE sessions is likely to significantly reduce the incidence of DBs.

**Conclusion:**

The findings of this study could serve as a valuable reference for decision-makers in organizing PE schedules, particularly in schools classified as “at risk”, where the prevalence of violence and incivility is above average.

## 1 Introduction

In the educational field, Disruptive Behaviours (DBs) are generally considered those behaviours that are likely to interfere, either directly or indirectly, with the teaching-learning process (1). The terms used in the literature to describe this type of behaviour can take different forms. It is therefore quite common to come across terms such as indiscipline, deviant behaviour or misbehaviour (2). However, what all these terms have in common is that teachers perceive the behaviour of one or more pupils as inappropriate to the context in which the action is taking place (3). Regardless of the subject being taught, DBs have a direct impact on the classroom climate and consequently on student performance (4). In PE, behaviour management is one of the main concerns of both novice and experienced teachers (5). DB not only disrupt the smooth running of the classroom, but is also considered to be a major source of stress for both teachers and students (6, 7).

The task turns to be more difficult in PE classes as the environment is different from the teaching conditions of other school subjects that use closed and restricted spaces (8). Indeed, PE classes ive rise to many DB episodes to occur, as pupils move around in larger spaces than in normal classrooms. Moreover, the implementation of motor actions that require pupils to interact with each other and with moving equipment and objects further complicates the teacher's task. In addition, PE is characterized by transitions between learning situations or breaks, and DB is proved to be significantly more frequent during these periods (9). Managing behaviour during these transitions is a challenging task for even the most experienced teachers (10, 11). All these factors potentially have a direct impact on the quality of learning and teaching. With this in mind, a growing number of researchers are investigating effective intervention methods and preventive behaviour management strategies to reduce the occurrence of DB episodes in students [e.g., (11, 12)].

In addition, several studies have shown that DB has become increasingly common in schools in recent years, in line with socio-economic changes. Older students report higher DB scores/frequencies and, consequently, the level most affected is secondary school and, to a lesser extent, primary school (1, 13, 14). In fact, for some time now, a growing number of teachers have confirmed that “students have changed”, that “students no longer have a taste for effort”, that “they no longer respect anything or anyone” and that “it used to be much easier to teach.." (15). In this sense, scientific studies on DB have classified several types of DB, ranging from non-compliance with teachers’ requests to aggressiveness (16, 17). Other researchers have used classifications of DB according to their severity. In this context, Brunelle et al. (18) developed a tool for observing disciplinary incidents specifically for PE, based on 3 levels of severity, ranging from a simple DB that has little influence on the course of the session (e.g., chattering) to a DB that requires the session to be interrupted (e.g., aggression).

DB may initially be mild or moderate, but if the teacher does not intervene effectively, DB may worsen (19, 20). If DB seems to be part of the PE teacher's daily routine, would it not be wiser to prevent it? With this in mind, several studies have attempted to identify strategies to prevent DB (8, 11).

In education, behaviour management can be approached from a number of different angles. However, this study approaches the issue from a different angle by attempting to establish a link between the occurrence of DB and the time of day. From the start, it is emphasized that few studies in the field of PE have dealt with this issue of school planning, especially when it comes to planning the daily time for PE in the school environment. Most studies deal with the organization of weekly or annual time. Blin and Gallais-Deulofeu (21) discuss the issue in terms of “school deregulation”. According to him, clowning around, gossiping, leaving classroom, distorting the activity, hurting pupils or breaking rules, all these manifestations of behavioral disturbances among pupils are numerous and varied, and are generally more pronounced at inappropriate time during the school day.

In the same way, research has shown that discipline management is directly linked to respecting the child's rhythm in the organization of school time. Testu (22) and Testu et al. (23) point out that there are good and bad times in the school day. During optimal periods, behaviour is more adapted to the school situation and vigilance and attention are high. In contrast, during suboptimal periods, these cognitive and behavioral capacities decline. Accordingly, research has shown that pupil behaviour that disrupts the smooth running of the classroom is directly linked to the planning and organization of the timetable. Hence the importance to consider the time of day, i.e., morning or afternoon (diurnal variations) or beginning, middle and end of the week (weekly variations).

Similarly, a number of studies have confirmed that mental skills are affected by the time of day (23–25). Montagner's (24) research concludes that the length and organization of the school day should be changed if schools are to give all children the best possible chance of success. In particular, new childcare policies need to be developed and school hours need to be reorganized. In this context, and because of the increase in the number of pupils with undisciplined behaviour, it may be that the incidence of DB is linked to the rhythm of the school day.

Furthermore, in PE, research has shown that the criteria that influence the organization of the subjects' timetable between the main and afternoon sessions are neither scientific nor rational (26). In fact, PE timetables are usually organized according to the availability of pupils, lessons in other subjects and available sports facilities, which means that PE can be practiced at any time of the day. However, a rational allocation of the time devoted to PE should take into account developments in sports management and school administration research (27, 28). Therefore, it seems clear that for some reason the school does not prioritize PE and does not really care about the potential impact of time of day variations on student behaviour in PE.

On the basis of all these considerations, the main aim of this study was to examine the effect of the organization of school time for PE on the occurrence of DB among pupils in lower secondary schools. Accordingly, this study was to answer the following question: does the timing of PE lessons influence the frequency of disruptive behavior?

## 2 Materials and methods

### 2.1 Participants and setting

After receiving a detailed explanation of the study's aims and procedures, a female PE teacher with 15 years of teaching experience accepted to participate. Observers were granted access to the classroom for the scheduled observations. Specific inclusion criteria were established to ensure the eligibility of participants. Initially, the selection process was based on the teacher's recommendations, as she nominated students who were identified as the most disruptive in her 10 classrooms. Three preliminary observation sessions were then conducted to validate the teacher's claims. A total of 349 pupils were observed during the pre-observation phase. The final inclusion criterion was defined as displaying a median level of disruption during at least 20% of the observation intervals.

**Figure 1:**
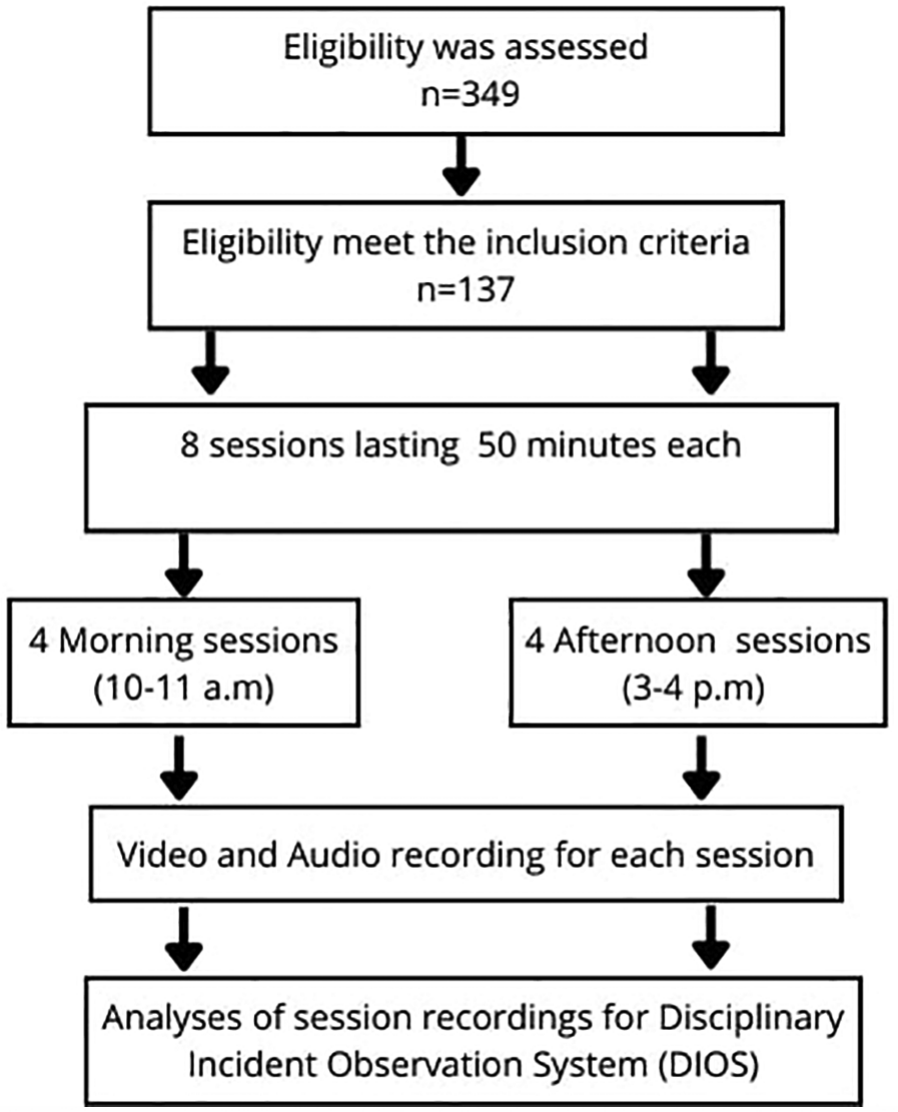
Flowchart of the study.

Participants were 137 Tunisian male students, whose data were included in the final analysis (Mean age = 13.63 ± 0.7 years; PE experience 5.7 ± 0.3 years) out of the initial 349 students who met the inclusion criteria based on preliminary observations (see Figure 1). All participants were in good health, and were selected from an underprivileged local region.

Working with the school administration and the participating teacher, the PE curriculum was standardized to ensure uniformity across all classes throughout the study. Specifically, the originally planned year 8 PE learning units were replaced with a single handball learning unit which extended from baseline phase to the end of the intervention phase. These adjustments were made to reduce the potential influence of confounding factors, such as variations in student motivation and lesson content, which could affect the dependent variable (i.e., student DB) across sessions.

Prior to the experiment, written informed consent was obtained from the students, clearly stating their agreement to participate in this study. In addition, the present study was conducted in accordance with the Declaration of Helsinki. The protocol was approved by the local research ethics committee (DBP: No. 147/2023).

### 2.2 Research design

The Influence of time is the dependent variable and the Disruptive behaviour serves as the independent variable in this study.

The observation focused on eight PE sessions, with the primary objective of recording the frequency of DB episodes among students. Recordings were made at two points during the day: the first in the morning (10–11 a.m.) and the second in the afternoon (3–4 p.m.). A systematic off-line observation was employed, utilizing a version of the Disciplinary Incident Observation System (DIOS) developed by Brunelle et al. (18). In order to assess the normal hour of going to sleep, a questionnaire was distributed to all participants, asking them to mention the time they go to bed. Based on their responses, showing that most of them sleep between 9 and 10 p.m. Our recommendations (sleeping before 10 p.m) were taken into consideration by all participants.

The data collected consisted of video and audio recordings of all the students' behaviour. The data was collected using two camcorders and a wireless microphone. The first camcorder was fixed in a corner of the field to capture the maximum amount of data from the learning situations. The observer with the second camcorder had the task of following the students as they moved around in order to record as many verbal exchanges as possible. The teacher was instructed to approach the students in case of any indiscipline on their part. The verbal exchanges of the teacher and the pupils close to him during the action were recorded by a microphone connected to the camcorder. A total of eight sessions of fifty (50) min each were filmed, giving a total of 400 min, which was subsequently used for analysis.

Time-lapse video analysis was employed, which involves watching the lesson several times in order to identify episodes of DB. The device used during the observation (“sound/image coupling”) allowed to compare the behaviour of the different actors and what they said (instructions, private or collective remarks, verbal and behavioural reactions of the teacher and the pupils).

### 2.3 Measure: disciplinary incident observation system (DIOS)

The DIOS is a direct observation instrument with predefined categories. Its purpose is to help the observer describe the content of disciplinary episodes that are likely to occur during PE lessons (18). This technique makes it possible to collect data on pupils' behaviours and teachers' reactions. A grid of categories of DBs allows the coder to record events by ticking the relevant categories in the chronological order in which they occur. This technique allows the observer to obtain the frequency of occurrence of each DB in the grid of predetermined categories. This system for observing disciplinary incidents is considered to be very robust in the literature and is frequently used in scientific documentation (9).

The DIOS presents eight categories: (1) The scenario of DB of the students, of which there are 19; (2) The number of students involved in the DB (i.e, the number of students involved in the situation); (3) the level of intensity (divided into three levels 1, 2 and 3); (4) the times when different DBs occur; (5) the threshold of accessibility of the DB by the teacher (i.e., did he or she notice the DB or not); (6) the types of teacher responses to the DB; (7) the impact of the DB adopted by the students on the progress of the lesson; (8) the impact of the teacher responses on the DB of the students. For the purposes of this study, only the first five categories that meet our objectives were selected.

DBs (19) are classified into three levels according to their severity. Level 1 (DB 1) includes DBs that generally have little impact on the classroom climate, but may still bother the teacher. Level 2 (DB 2) includes moderate DBs that are likely to disrupt the smooth running of the class in the short to medium term. Level 3 DBs (DB 3) severe manifestations disrupt the smooth running of the class and require immediate intervention.

In the DIOS framework, a DB is classified as accessible if it occurs within the teacher's field of vision or can be audibly perceived. Conversely, a DB is considered inaccessible if it is not visible or audible to the teacher. The DIOS proposes ten moments that make it easier to identify DB. These moments are grouped according to the order in which the different phases of a PE session take place. Moments 2–5 concern the preparation phase (handling and warming up). They are followed by moments 6 and 7, which are the realization phase (the body of the session). Moments 8 and 9 are the integration phase (return to rest and feedback).

In order to facilitate the task of observation and minimize disruption of students, the behaviours demonstrated before and after the lesson, which appear in the DIOS grid, were not taken into account. Thus, the observation starts at the handling phase, the moment when the teacher starts the lesson by receiving the students online to take attendance, and concluded just after the return to the calm phase, during the final feedback session and class dismissal.

### 2.4 Coding method

The coder was trained in the use of DIOS for coding video recordings. Firstly, the coder worked as a team to become familiar with the observation grid and to master all its components. It was necessary to practice classifying the DBs that occur during PE lessons. Secondly, individual coding was followed by the comparison of the grids, which highlighted some discrepancies.

It was therefore sometimes necessary to go back to the definitions of the components of the grid in order to ensure the conformity of the DBs and to agree on the same interpretation. However, there was still room for subjectivity.

After the training period, the coding of the two coders was subjected to a reliability test several times before the final coding began. At the start of the training, the intercoder reliability rates were between 60% and 70%. Towards the end of the training phases, the reliability rate finally reached 85% for the identification of DBs and their classification.

### 2.5 Data analyses

A matrix corresponding to the various categories retained from the DIOS observation grid enabled the research data to be entered and transferred to an Excel file, which was then transferred to an SPSS file (IBM SPSS Statistics, French version 20) for the statistical analyses required to achieve the study's objectives.

The analysis procedure consisted firstly in counting the absolute and relative frequencies of DB among students at two times of the day (morning and afternoon). The non-parametric chi-square test was used to compare the frequencies of occurrence of DB. Chi-square is a wide-range hypothesis test that measures the relationship between two categorical variables using the contingency coefficient and Cramer's V (Stafford & Bodson, 2006: in (29). To compare the means of the DB observed between the morning and the afternoon, the Student t-test (for sample paired) was used when normality was verified (>0.05), the non-parametric test, as well as Wilcoxon paired sample test, when the distribution did not meet normality (<0.05).

## 3 Results

### 3.1 Descriptive analysis of the frequency of occurrence of DB for the whole sample during the morning (10 am to 11 am) and afternoon (3 pm to 4 pm)

The data in Table 1 indicate the frequency of DBs in each session. In the morning, 160 DBs were observed across the four sessions, averaging 40 DBs per session, or 0.8 DBs per minute. The afternoon sessions showed 97 DBs across the same number of sessions, averaging 24.25 DBs per session, or 0.485 DBs per minute. Out of the 19 DB types identified in the DIOS, 15 were recorded in this study, with the remaining four not observed: (1) leaving the room; (2) deliberately breaking the rules; (3) behaving dangerously; and (4) refusing instructions.

**Table 1:** Frequency of types of DB (morning and afternoon).

Disruptive Behaviours (DBs)	Morning (10 am to 11 am)		Afternoon (3 pm to 4 pm)	
	Effectif (%)		Effectif (%)	
Level 1	50	31.3%	31	32.0%
1. Is distracted	4	2.5%	4	4.1%
2. Chatting	40	25%	24	24.7%
3. Arriving late	4	2.5%	0	0.0%
4. Unkempt	2	1.3%	3	3.1%
Level 2	80	50%	59	60.8%
5. Clown around	24	15.0%	16	16.5%
6. Bickering	4	2.5%	0	0.0%
7. Harasses	6	3.8%	18	18.6%
8. Makes noise	35	21.9%	12	12.4%
9. Distorts the activity	11	9.9%	9	9.3%
10. Stops practising	0	0.0%	4	4.1%
Level 3	30	18.8%	7	7.2%
11. Critical	10	6.3%	2	2.1%
12. Attacks equipment	3	1.9%	2	2.1%
13. Attacks	0	0.0%	1	1.0%
14. Is rude	4	2.5%	0	0.0%
15. Ridicules	13	8.1%	2	2.1%
Total	160	100%	97	100%

Table 2 shows that there was a significant difference in the averages of DB observed between the morning and afternoon sessions (*p* = 0.008 < 0.05). These results show that the time of day when PE is practiced has an impact on the appearance of DB in pupils. It seems that planning the time of the PE session in the afternoon has a positive effect on reducing DB.

**Table 2:** Comparison of DBs observed among pupils between morning and afternoon sessions.

DBs	Morning (10 am to 11 am)	Afternoon (3 pm to 4 pm)		
	Average (±SD)	Average (±SD)	*t*	Sig (p)
Total	40 ± 2.944	24.25 ± 2.062	6.311	.008*

*p < 0.05.

The counts of DBs (level 1, 2 and 3) during the morning and afternoon showed a considerable decrease. In fact, the results of the Student's t-test (paired samples) showed a significant difference in the means of DB1 and DB3 observed between the morning and afternoon in relation to severity level 1 and 3 (respectively: *t* = 6.333; *p* = 0.008 < 0.05 and *t* = 6.734; *p* = 0.007 < 0.05). However, although the results show a remarkable decrease for level 2 DBs (See Table 3) between morning and afternoon, this decrease does not reach the significance threshold (*p* = 0.08 > 0.05).

**Table 3:** Breakdown of DBs.

DBs	Morning (10 am to 11 am)	Afternoon (3 pm to 4 pm)		
	Average (±SD)	Average (±SD)	*t*	Sig (p)
DB 1	12.5 ± 1.732	7.75 ± 0.957	6.333	.008*
DB 2	20 ± 2.16	14.75 ± 2.63	2.605	.08
DB 3	7.5 ± 2.082	1.75 ± 1.258	6.734	.007*

*p < 0.05.

**Figure 2:**
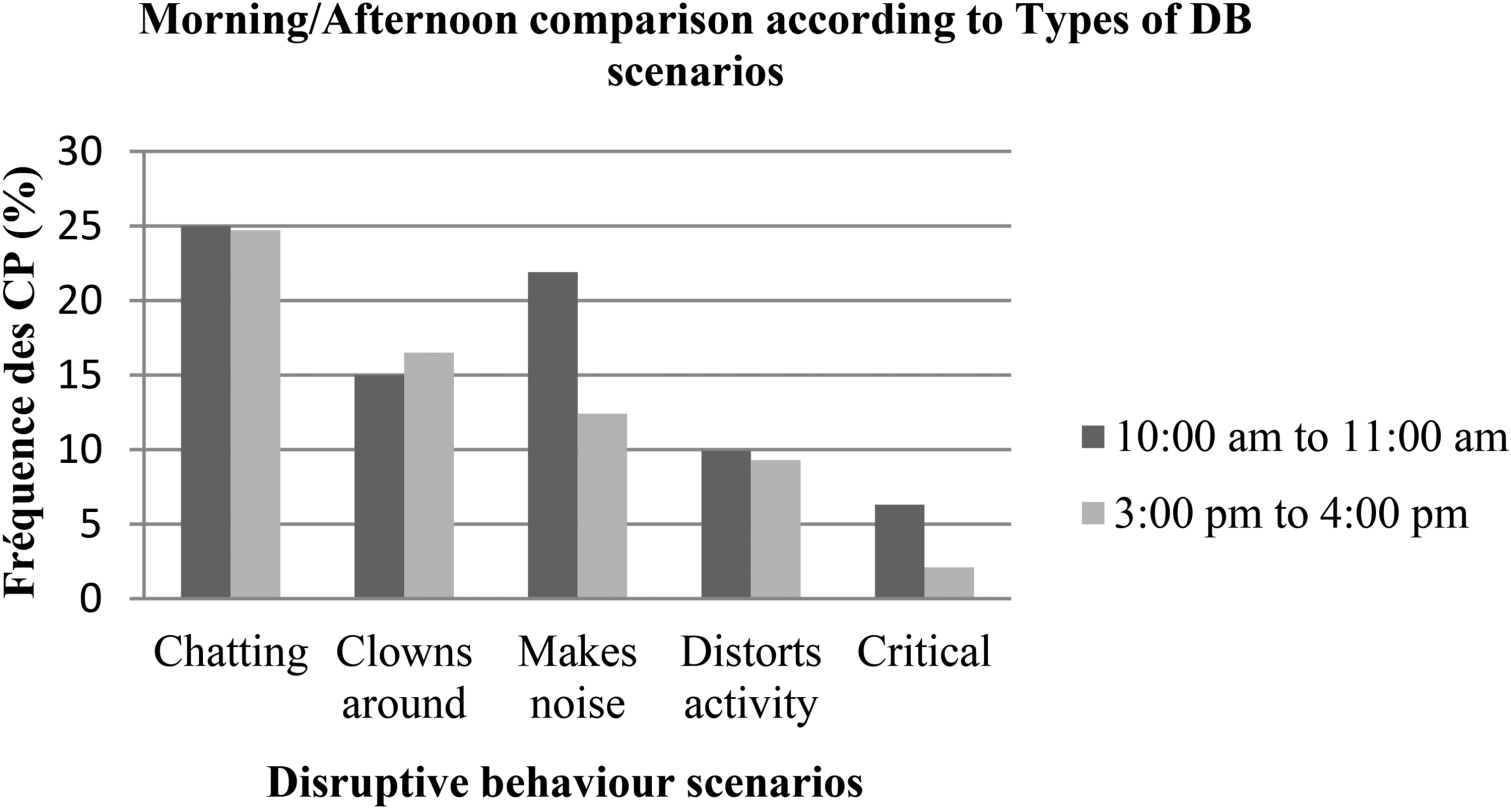
Different forms of DB during PE sessions (morning/afternoon).

Figure 2 below illustrates the five dominant DBs observed during the morning and afternoon PE sessions. In fact, the results show that the most marked DBs decreased during the afternoon sessions, so we can conclude that the time of day of PE has a significant influence on the frequency with which DBs appear.

### 3.2 DBs according to different PE session phases

**Figure 3:**
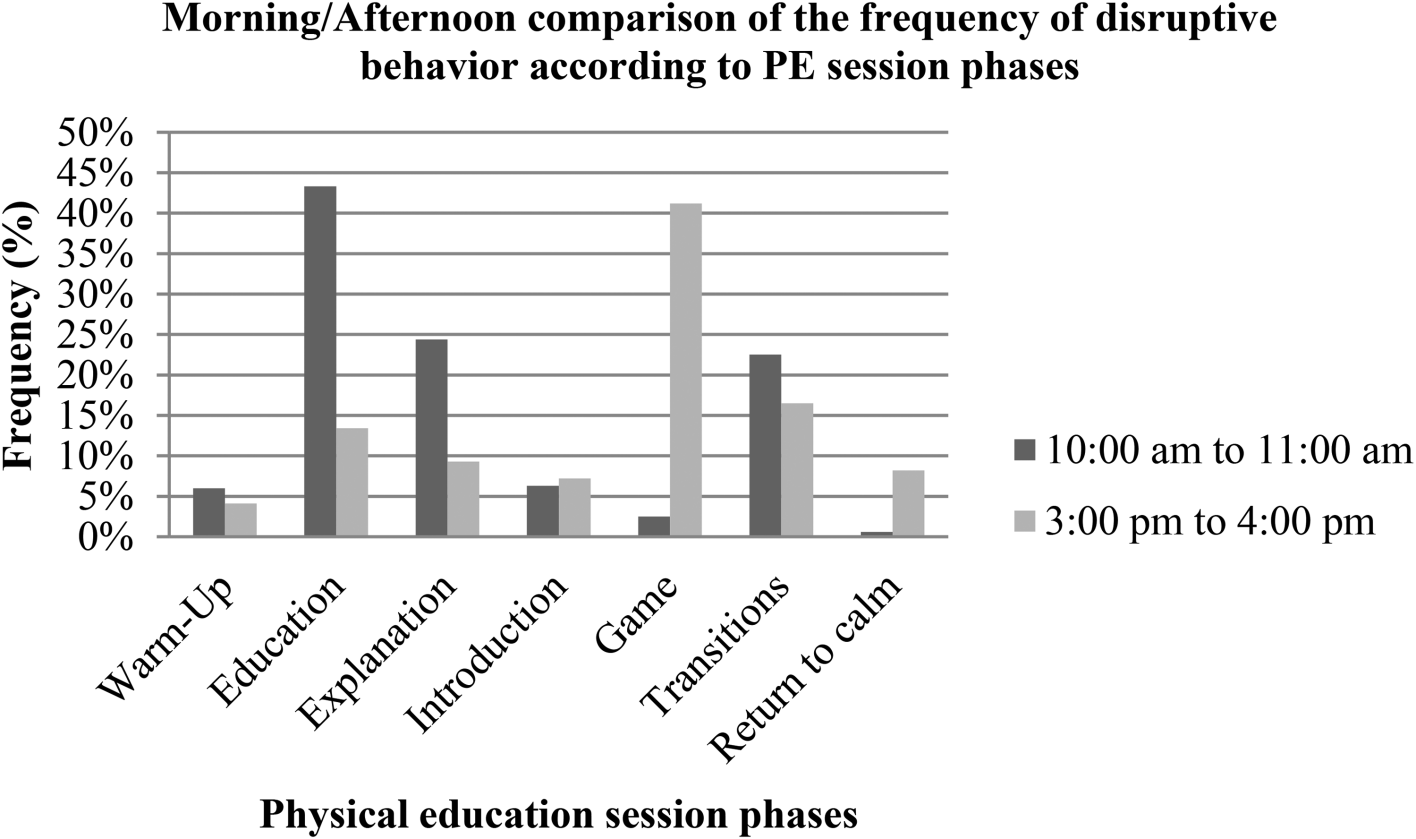
Frequency of DB across phases of the PE session (morning/afternoon).

Figure 3 below illustrates this relationship between the frequency of occurrence of DBs according to the different phases of the PE session.

A comparison between the two times of day using Chi-square shows a significant relationship between the time of day and the frequency of appearance of DBs according to the different phases of the session: kh2 = 91.999; ddl = 6; *p* = 0.000 (VCramer = 0.598; C = 0.513 at *p* < 0.05).

## 4 Discussion

For several decades, school rhythms have been a key topic of reflection for curriculum designers, educators, institutional decision-makers, chronobiologists and chronopsychologists. According to Leconte (30), in a review of the literature on the reorganization of school rhythms, it is recommended that the first priority should be the school day. The author emphasizes that “ *it is only when this period is understood that the other times can be changed*”. In this sense, the author suggests respecting pupils’ time availability as much as possible and cites the findings of Bianco and Bressoux (31), which clearly show that the best results are achieved in classes where the teacher is able to modulate his functioning throughout the day according to what he sees from his pupils.

The aim of this study was to measure the frequency of DB in PE at two different times of the day, morning and afternoon. The main findings revealed that conducting physical activity during PE in the afternoon is likely to significantly reduce the occurrence of DBs.

Consistent with the literature, the results of the current study showed that pupils were less likely to engage in DB in the afternoon than in the morning. In fact, the results of our study showed that the average number of pupils likely to engage in DB was lower in the afternoon than in the morning, although this reduction in the number of pupils did not reach the threshold of significance. More pupils were involved in physical activities in the morning (M = 2.51 ± 1.52) than in the afternoon (M = 1.98 ± 1.12). This outcome aligns with what researchers in chronopsychology describe as the peak time for psychotechnical and attentional performance, which typically occurs in the afternoon (23). As a result, students are more alert and perform better in physical activities during the afternoon. It's also noteworthy that the French Ministry of Education and Youth, following a study conducted in 2010/2011, reported that organizing PE sessions in the afternoon had positive effects on students' behaviour and mood. Therefore, our results seem to be in line with other research on the fact that pupils feel better in the afternoon in terms of practicing PSA and taking part in physical activity.

Furthermore, our findings align with other researches showing that peak cognitive performance in the afternoon leads for better productivity. In fact, this seems to be related to circadian driven fluctuations in core body temperature and cortisol levels. For example, Schmidt et al. (32) found that the reaction time increased by 10% in the afternoon compared to the morning. On another note, our results coincide with Baehr et al. (33) research, who tied afternoon exercise to improve better motor skill retention, which we belive is related to better engagement, as mentioned in our study results. Additionally, our findings showed less DB in the afternoon, which we suggest resulted in better attention, leading to improved performance. This is of interest and was further demonstrated by Schmidt et al. (32), who reported that the time of day effects attention and advocated for aligning tasks with circadian peaks.

Moreover, the results of our research reported a significant relationship between the frequency of occurrence of DB during the PE session and the time of day. In fact, the results showed a significant reduction in DB between the morning and the afternoon, particularly during teaching, explanation and transition moments. Consequently, our findings suggest that pupils are more attentive in the afternoon than in the morning. These results confirm other studies on this topic (34, 35). Thus, among the moments in PE that are likely to generate DB, the time devoted to explanations, transitions, educational activities and games are potentially the most important (9). These moments require a particularly high level of attention from pupils. Thus, the periods of the lesson devoted to instruction, are well as those devoted to practicing skills of a technical or tactical nature, are moments that require a great deal of attention from the pupils, who need to concentrate. Explanation time, when students receive instructions regarding learning situations or organizational aspects, is also critical for maintaining attention. Additionally, transitional periods which are especially fragile moments, can easily disrupt students' focus and lead to DB. According to Nault and Lacourse (36), transitions are opportunities to break students' concentration. Therefore, to ensure smooth transitions, students' concentration and attention must be at an optimal level.

With this in mind, it seems possible to establish a three-dimensional relationship between pupils' attention, the times during the session when DB is most likely to occur, and the time of day. Research has shown that pupils' attention varies, particularly in the afternoon after 3 pm (30). The afternoon, therefore, represents the “peak of the day”, when students are likely to be most attentive (23, 37). The “potentially strong” moments of the session require a high level of attention from the students. According to Desbiens et al. (9), these times are more likely to generate DB than other times of the day as classified by Brunelle et al. (18).

Based on this three-dimensional relationship, and considering the results of this study showed that the manifestation of DB was significantly less in the afternoon than in the morning, according to the time of the session, it seems logical to conclude that students who participate in physical activity have better attention in the afternoon than in the morning. Our findings align with chronopsychological research, which indicates that the afternoon, specially the peak period from 4 p.m. onwards, is when optimal levels of attention and vigilance are achieved (24, 38). This period is also the time when physical activity and sport are recommended (39).

In addition, studies by Sue and Cassia (40) showed that the reorganizations of school rhythms has a positive impact on the development of a range of skills in pupils, including listening skills, communication skills, respect for others, mutual support and compliance with rules and instructions. Based on numerous observations, the authors also report that the reorganization of school rhythms helps to develop relationships between children outside the rules of the classroom, in small, mobile groups that from during the course of activities. Pupils were more likely to see school as a place where they could meet their friends. These findings strongly support our own, which also point to the beneficial effects of reorganizing school rhythms on pupils' behaviour and mood, particularly in terms of reducing the frequency of DB. In this context, Sue and Cassia (40) reported that the reorganization of school rhythms led to a reduction in violence and incivility, particularly within the school.

Like all research, our study has limitations. First, the sample size is relatively small compared to some studies in the literature, which may limit the generalizability of the results. Given the importance of the topic, teamwork would be appreciated. However, since that the majority of schools do not incorporate napping into their schedules, which considered an unusual practice (41), it is unlikely that this factor influenced the results of our study. These limitations provide an opportunity to expand future research in this area. As, it would also be interesting to examine the effect of the time of day on other variables involved in the teaching-learning process.

Future studies could investigate the hormonal mechanisms underlying the link between afternoon PE sessions and a decrease in DB, particularly in teenagers going through pubertal and circadian shifts. Specific function as cortisol and dopamine, two key neuroendocrine markers involved in stress management, attention, and behavioral control, could receive particular focus. Furthermore, examining how the timing of PE classes affects behavioral outcomes and academic achievement may provide valuable insights into the interdisciplinary benefits of chronobiologically based scheduling. It's important to highlight the need for further studies with female participants, which could contribute to a more comprehensive understanding of circadian rhythms and their influence on DB.

## 5 Conclusion

Our results showed that physical activity during afternoon PE sessions was likely to reduce the incidence of Disruptive Behaviour (DB). These findings are consistent with those found in the literature. In fact, several sources point to the positive effects of afternoon physical activity. These positive effects include a decrease in violence and incivility (40). The results of our research could be a source of inspiration for decision-makers in planning the time devoted to Physical Education (PE), especially in schools identified as “at risk”, where the incidence of violence and incivility is higher than average.

## Data Availability

The raw data supporting the conclusions of this article will be made available by the authors, without undue reservation.
